# Prevalence of the association of vulvar lichen sclerosis with Hashimoto’s thyroiditis^⋆^

**DOI:** 10.1016/j.abd.2023.04.007

**Published:** 2024-03-22

**Authors:** Marcela Scárdua Sabbagh de Azevedo, Antônio Chambo Filho, Lucia Martins Diniz, July Barcellos Quimquim, Vickie White Loureiro Souza, Luana Amaral de Moura

**Affiliations:** a Hospital Universitário Cassiano Antônio Moraes, Universidade Federal do Espírito Santo, Vitória, ES, Brazil; b Universidade Federal de Minas Gerais, Belo Horizonte, MG, Brazil; c Department of Gynecology and Obstetrics, Escola Superior de Ciências da Santa Casa de Misericórdia de Vitória, Vitória, ES, Brazil; d Universidade Federal do Rio de Janeiro, Rio de Janeiro, RJ, Brazil; e Department of Internal Medicine (Dermatology), Universidade Federal do Espírito Santo, Vitória, ES, Brazil; f Dermatology, Hospital da Santa Casa de Misericórdia de Vitória, Vitória, ES, Brazil; g Gynecology and Obstetrics, Instituto de Assistência Médica ao Servidor Público Estadual de São Paulo, São Paulo, SP, Brazil

Dear Editor,

Lichen sclerosis (LS) is a chronic inflammatory and cicatricial disease of unknown etiology, which mainly affects the anogenital region of women in the pre-pubertal and post-menopausal period.^1^, ^2^, ^3^ Its actual prevalence remains unknown, mainly due to underdiagnosis.^4^ The absence of symptoms is observed in up to 39% of cases and the omission of anogenital complaints by female patients contribute to this scenario.^4^

Clinically, vulvar lichen sclerosis (VLS) manifests as erythematous papules or ivory patches, sometimes hyperkeratotic, which can coalesce to form plaques. Overall, the lesions are symmetrical and affect the labia minora and majora, the perineum and the skin of the perianal region. In more advanced stages, there may be effacement of the labia minora and clitoris. Pruritus, pain and dyspareunia are among the most frequently reported symptoms and tend to worsen at night.^2^, ^4^ Dysuria, urinary dysfunction and urinary bleeding due to fissures may also occur.^2^, ^5^ Histopathology shows atrophy of the epidermis, hyperkeratosis and basal cell degeneration. Dense fibrosis, edema and chronic perivascular inflammation can be observed in the papillary dermis, with a predominance of eosinophils.^2^

The etiology of VLS is yet to be fully elucidated, but there is growing evidence of a multifactorial origin, including genetic, autoimmune, hormonal aspects and infectious history.^2^ The involvement of autoimmunity has been proposed by the frequent association between VLS and a personal or family history of autoimmune diseases.^4^ Tissues affected by VLS show dysfunction of T-regulatory cells (Tregs), and low levels of interleukin-10, creating an environment favorable to autoimmunity.^4^, ^6^ This hypothesis is strengthened by the detection of autoantibodies in the serum of patients with VLS, such as antibodies against extracellular matrix protein 1, found in nearly 74% of cases.^1^, ^7^ Moreover, antibodies against members of the basement membrane zone, especially the transmembrane proteins BP180 and BP230, have been described in 30% of patients with VLS, without correlation to clinical severity and pruritus.^7^, ^8^ Up to 40% of patients with VLS have the NC16A domain of BP180 as a target for circulating T cells.^7^ Recently, a case was described of a 77-year-old woman, previously diagnosed with VLS and localized scleroderma (LS), who developed bullous pemphigoid, with lesions manifesting exclusively in areas previously affected by VLE and LS. The authors believe that pre-existing VLS and LS acted as facilitators in the development of bullous pemphigoid, due to the reactivity of T-cells to BP180, often associated with VLS, and the increase in Th2 signaling associated with LS.^7^

Thyroid diseases are the autoimmune disorders most frequently associated with VLS, present in up to 39% of cases.^4^, ^9^ Hashimoto’s thyroiditis (HT) is characterized by thyroid hyperplasia, infiltration of lymphocytes in the glandular parenchyma and presence of antibodies against thyroid antigens. It is considered the main cause of hypothyroidism in Brazil and affects around 2% of women worldwide.^10^ Several authors support the association between VLS and autoimmune thyroid diseases, with autoimmunity being the likely link between these conditions.^1^, ^2^, ^4^

The measurement of thyroid-stimulating hormone (TSH) is considered the reference testing for the detection of hypothyroidism. When elevated, it suggests gland hypofunction, and the measurement of free thyroxine (FT4) is indicated to differentiate overt hypothyroidism (low FT4) from subclinical hypothyroidism (normal FT4). The presence of anti-thyroid peroxidase (anti-TPO) and/or anti-thyroglobulin autoantibodies identifies Hashimoto’s thyroiditis.^10^ Anti-TPO positivity is the most important characteristic of HT, present in approximately 95% of patients, in addition to having a sensitivity of 90% in the diagnosis of autoimmune thyroid diseases.^10^

VLS attracts the attention of dermatologists and gynecologists, largely due to the enormous damage it inflicts on patients quality of life, in addition to favoring the emergence of squamous cell carcinoma (SCC).^4^

A prospective observational study was conducted at a University Hospital. During two years, 63 women with typical signs and symptoms of VLS were selected at the Vulvar Diseases Clinic and histopathological confirmation was subsequently carried out. For the investigation, TSH, FT4 and anti-TPO were requested, and patients with elevated TSH levels (above the upper limit of the reference value), associated with reduced FT4 (below the lower limit of the reference value), and positive anti-TPO were considered to have HT. All patients with a previous diagnosis of hypothyroidism were excluded from the study.

The project was approved by the Ethics Committee of the University Hospital on 07/31/2018, under CAAE number: 92818418.8.0000.565. For the statistical analysis of data, the chi-square test and confidence interval (OR) were used, considering a p≤0.05 as statistically significant.

Sixty-three patients were included in the study, 27 (42.8%) white and 36 (57.2%) non-white (brown or black), in contrast with other studies in which the largest number of patients in the sample comprised Caucasian patients, making it impossible to generalize the results to other ethnicities.^9^, ^11^ The average age was 59 years, ranging between 33 and 87 years (Fig. 1), corroborating the data from Cooper et al. and Kreuter et al., who recorded a mean age of 63 and 49 years, respectively.^4^, ^5^, ^9^, ^11^

**Figure 1:**
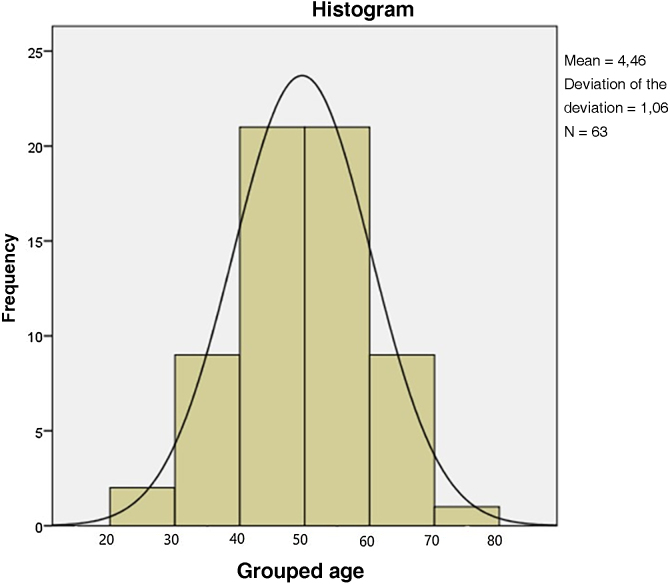
Age distribution of the 63 patients with VLS.

Of the analyzed patients, 16 (25.4%) had Hashimoto’s thyroiditis, a higher frequency than that found in the general population, but in agreement with the literature on patients with VLS. However, the frequency found is slightly higher than in studies with a large sample.^1^, ^2^ Kreuter et al. analyzed 532 patients, of which 82 (15.4%) had one or more autoimmune diseases, highlighting the prevalence of 65 (12.2%) patients with LS and autoimmune thyroid disease (HT or Graves’ disease), of which 60 were women.^11^ In this study, of the 396 female individuals, 322 (81.3%) had LS in the anogenital region. Cooper et al. when investigating 190 women with VLS, demonstrated that 28.4% had at least one autoimmune disease; and 16.3% had VLS associated with autoimmune thyroid disease, contrasting with 9% in the healthy control group (p < 0.001).^9^

In the present study, the grouping of VLS and HT patients according to age showed that seven of them (43.8%) were up to 59 years old and nine (56.3%) were 60 years old or older, with no statistical difference (p = 1.000) ‒ Fig. 2. When divided by ethnicity, nine patients (56.2%) were white and seven (47.8%) were of another ethnicity, statistically without significance (p = 1.714), as shown in Table 1.

**Figure 2:**
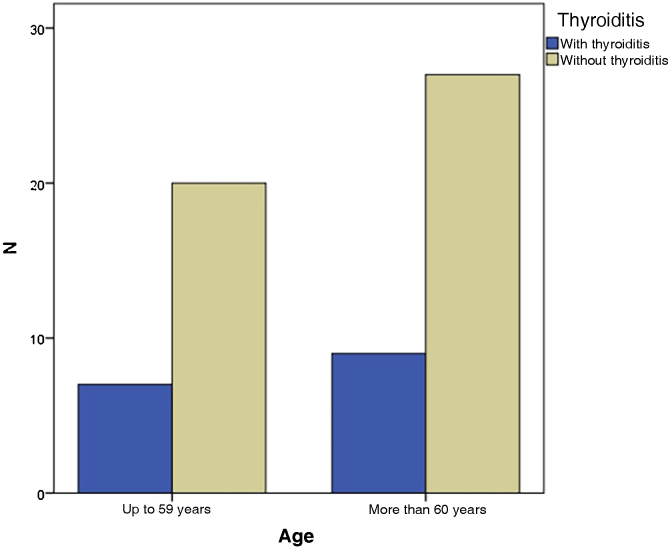
Distribution of patients with VLS in relation to age, according to whether the thyroid is affected. There was no statistical significance (p = 1.000).

**Table 1:** Relationship of VLS patients and thyroiditis with the variables age, skin color and state procedence.

		Thyroiditis					
		Yes		No			
		n	%	n	%	p	OR
**Age**	Up to 59 years	07	43.8	20	42.6	1.000	1.037 (0.422‒2.432)
	60 years or older	09	56.2	27	57.4		
**Skin color**	White	09	56.2	18	38.3	0.251	1.714 (0.731‒4.021)
	Non-white	07	47.8	29	61.7		
**State**	Espírito Santo	12	75.0	40	85.1	0.286	0.635 (0.251‒1.602)
	Others	04	25.0	07	14.9		
**Total**		16	100	47	100		

The limiting factors of the study were the small sample size and the absence of a control group. However, the obtained data corroborate the association of VLS and autoimmune thyroid diseases. Of the 63 evaluated patients with VLS, 16 (25.4%) were diagnosed with HT. Patients age and ethnicity did not have a statistically significant impact on the diagnosis. It is believed that the frequent coexistence of these diseases is related to autoimmune dysregulation, possibly common to both. We have not found studies to date that specifically correlate VLS and Hashimoto’s thyroiditis. The analyses indicated that the impact of screening for autoimmune diseases, especially, HT in individuals with VLS is greater than previously suggested.

## Financial support

None declared.

## Authors’ contributions

Marcela Scárdua Sabbagh de Azevedo: Design and planning of the study; drafting and editing of the manuscript; collection, analysis and interpretation of data; critical review of the literature; approval of the final version of the manuscript.

Antônio Chambo Filho: Design and planning of the study; collection of data, or analysis and interpretation of data; collection, analysis and interpretation of data; effective participation in research orientation; intellectual participation in the propaedeutic and/or therapeutic conduct of the studied cases; approval of the final version of the manuscript.

Lucia Martins Diniz: Drafting and editing of the manuscript or critical review of important intellectual content; effective participation in research orientation; critical review of the literature; approval of the final version of the manuscript.

July Barcellos Quimquim: Design and planning of the study; collection of data, or analysis and interpretation of data; collection, analysis and interpretation of data; approval of the final version of the manuscript.

Vickie White Loureiro Souza: Design and planning of the study; collection of data, or analysis and interpretation of data; collection, analysis and interpretation of data; approval of the final version of the manuscript.

Luana Amaral de Moura: Drafting and editing of the manuscript or critical review of important intellectual content; effective participation in research orientation; critical review of the literature; approval of the final version of the manuscript.

## Conflicts of interest

None declared.
